# Potential Role of Oral Rinses Targeting the Viral Lipid Envelope in SARS-CoV-2 Infection

**DOI:** 10.1093/function/zqaa002

**Published:** 2020-06-05

**Authors:** Valerie B O’Donnell, David Thomas, Richard Stanton, Jean-Yves Maillard, Robert C Murphy, Simon A Jones, Ian Humphreys, Michael J O Wakelam, Christopher Fegan, Matt P Wise, Albert Bosch, Syed A Sattar

**Affiliations:** 1 Systems Immunity Research Institute; 2 School of Medicine; 3 School of Dentistry; 4 School of Pharmacy and Pharmaceutical Sciences, Cardiff University, CF14 4XN, UK; 5 Department of Pharmacology, University of Colorado Denver, Aurora, CO 80045, USA; 6 Babraham Institute, Babraham Research Campus, Cambridge, CB22 3AT, UK; 7 University Hospital of Wales, Cardiff, CF14 4XW, UK; 8 Enteric Virus Laboratory, University of Barcelona, 08028 Barcelona, Spain; 9 Faculty of Medicine, University of Ottawa, Ottawa, Ontario K1H 8M5 Canada

**Keywords:** coronavirus, lipid, envelope, oropharynx, virus, respiratory

## Abstract

Emerging studies increasingly demonstrate the importance of the throat and salivary glands as sites of virus replication and transmission in early COVID-19 disease. SARS-CoV-2 is an enveloped virus, characterized by an outer lipid membrane derived from the host cell from which it buds. While it is highly sensitive to agents that disrupt lipid biomembranes, there has been no discussion about the potential role of oral rinsing in preventing transmission. Here, we review known mechanisms of viral lipid membrane disruption by widely available dental mouthwash components that include ethanol, chlorhexidine, cetylpyridinium chloride, hydrogen peroxide, and povidone-iodine. We also assess existing formulations for their potential ability to disrupt the SARS-CoV-2 lipid envelope, based on their concentrations of these agents, and conclude that several deserve clinical evaluation. We highlight that already published research on other enveloped viruses, including coronaviruses, directly supports the idea that oral rinsing should be considered as a potential way to reduce transmission of SARS-CoV-2. Research to test this could include evaluating existing or specifically tailored new formulations in well-designed viral inactivation assays, then in clinical trials. Population-based interventions could be undertaken with available mouthwashes, with active monitoring of outcome to determine efficacy. This is an under-researched area of major clinical need.

## The Viral Lipid Envelope

**Figure 1.**
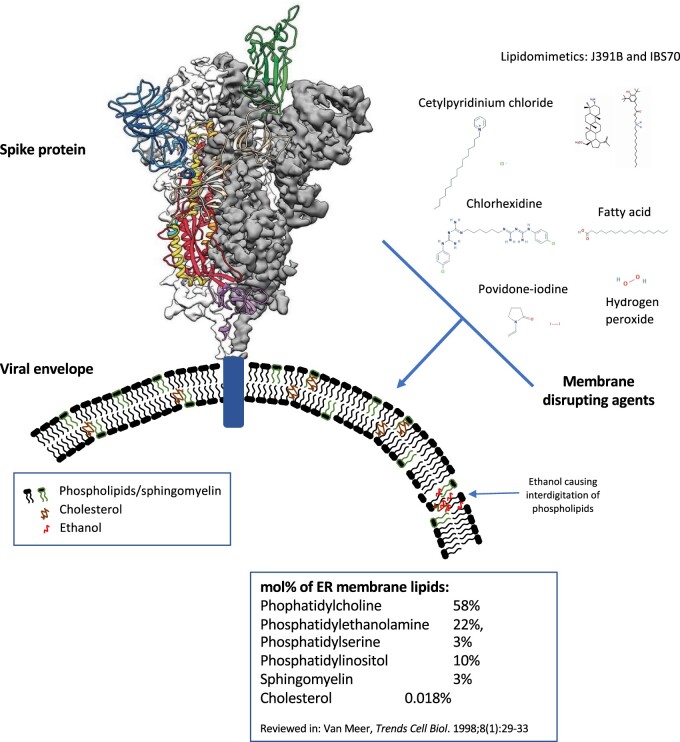
Cartoon Representation of the SARS-CoV-2 Glycoprotein, Embedded in the Viral Envelope, along with Membrane Disrupting Agents. Ribbon diagram was obtained from Wrapp et al.,^1^ chemical structures were from PubChem (https://pubchem.ncbi.nlm.nih.gov/) and Nieto-Garai et al.^2^

In common with many viruses, such as influenza and herpes simplex, coronaviruses are surrounded by a fatty layer, called a “lipid envelope,” into which the spike glycoproteins required for infection are inserted (Figure 1). Viral envelopes are acquired at host cell membranes—some at the plasma membrane, others at internal cell membranes such as the nuclear membrane, endoplasmic reticulum, and Golgi complex.^3^^,^^4^ During this, viral proteins are incorporated at the expense of host cell proteins, creating the shed viral particle.^5^ Thus, for most viruses, the envelope lipids are considered to be the same as the host membranes (phospholipids, sphingolipids, and some cholesterol). Lipid composition is not the same across subcellular membranes with mammalian plasma membranes having higher cholesterol and sphingolipid content.^6–12^ While the lipid makeup of the envelope of SARS-CoV-2 has not been characterized yet, coronaviruses are known to bud from the endoplasmic reticulum Golgi intermediate compartment (ERGIC), before being transported by exocytosis in cargo vesicles.^13^^,^^14^ This indicates their composition will be related to endoplasmic reticulum membrane, which contains more phosphatidylcholine, but less cholesterol and sphingolipids than the plasma membrane.^6–12^ A recent report demonstrated that coronavirus (HCoV-229E) regulates host lipid metabolism in response to infection, in common with many other viruses.^15–17^ However, no information on the virus lipid envelope composition was provided, and its specific composition has not been determined experimentally.

## The Soap/Alcohol Virucidal Public Health Advice Relating to Surface Neutralization

It is widely known that interfering with the lipid envelope represents a virucidal strategy to target many coronaviruses, with a large body of work evidencing the impact of many agents.^18^^,^^19^ For a summary, refer to Kampf et al., a systematic review providing tables showing data from different original publications for inactivation of coronaviruses by biocidal agents in suspension tests.^19^ During the 2003 SARS-CoV outbreak, viral material was detected on hospital surfaces, leading to the idea that surface decontamination would be an important approach. At that time, various compounds were considered, including ethanol at high concentrations of 60%–70% (v/v), since these doses had been found to be highly effective against several viral pathogens, including coronaviruses.^19–21^ Recent studies on SARS-CoV-2 also support this, with high concentrations being highly effective as shown in a recent preprint.^22^ The historical reason for only testing high concentrations in microbicidal research has been that these give broad-spectrum activity toward bacteria, viruses, and fungi, and thus for use on inanimate surfaces/fomites where the target microbes are unknown would be always preferred. The consensus view is that enveloped viruses, such as herpesviruses, orthomyxoviruses, paramyxoviruses, and coronaviruses are highly sensitive to 60%–70% (v/v) ethanol with almost immediate inactivation, while nonenveloped viruses are less or not susceptible.^19–22^

We are now widely encouraged to use soap or 60%–70% alcohol-based gels to inactivate SARS-CoV-2, based on the view that these agents damage the lipid envelope, including in recent World Health Organisation (WHO) and Environmental Protection Agency recommendations (https://apps.who.int/iris/bitstream/handle/10665/331138/WHO-WPE-GIH-2020.1-eng.pdf; https://www.epa.gov/pesticide-registration/list-n-disinfectants-use-against-sars-cov-2; https://www.who.int/gpsc/tools/GPSC-HandRub-Wash.pdf) . At the same time, there has been no discussion of oral antiviral strategies, apart from a recent response to an article in the *British Medical Journal* calling for protection for healthcare workers against infection (https://www.bmj.com/content/369/bmj.m1324/rr-5). Properly designed clinical trials that address this issue are currently lacking in the literature. Current WHO interim guidance on clinical management of SARS-CoV-2 in the home is focused on the use of personal protection, including face masks, along with hand, clothing, and surface sanitation, to reduce risk of airborne and direct spread of the virus, but does not mention oral hygiene (https://apps.who.int/iris/handle/10665/331133). Thus, its utility in the setting of SARS-CoV-2 has not been considered systematically, and there is a lack of either positive or negative robust clinical evidence.

Mouthwashes vary widely in composition; however, some commercially available formulations contain ethanol at 14%–27% (w/v) in the United Kingdom, Europe, and the United States. Prompted by this, we reviewed the available scientific literature to establish whether oral treatment using ethanol-based or other types of mouthwashes could present a strategy to either dampen or reduce viral load, to potentially restrict virus transmission in the current pandemic situation, particularly for vulnerable individuals or healthcare workers. We found that there is a paucity of data systematically testing the impact of lower (less toxic) ethanol concentrations on enveloped virus inactivation, with most simply reiterating the use of the higher concentrations described above.^18^^,^^20^^,^^21^^,^^23–26^ We also found a paucity of robust clinical studies in this area that address in a randomized double-blind manner the impact of oral rinsing on objective measures, specifically neutralization of enveloped viruses, including coronaviruses.

## Viral Load, Saliva/Throat Virus, and Disease Severity in SARS-CoV-2

It is becoming increasingly recognized that the throat is a major site of replication and shedding of virus in COVID-19 illness, and that viral load is important.^27^ Throat and sputum are abundant in particles, which peak 5–6 days after symptom onset, and decline thereafter.^28^^,^^29^ Viral load correlates with older age,^29^ and a study of 76 patients in Nanchang, China, showed that those with severe SARS-CoV-2 tend to have higher viral load and longer virus-shedding period than those with mild disease.^30^ Similarly, viral load was linked with lung disease severity in a study of 12 patients with pneumonia.^31^ Many asymptomatic individuals have modest levels of detectable viral RNA in their oropharynx for at least 5 days, which is similar to individuals with clinical symptoms.^32^ Data from GTEX gene expression data indicate that angiotensin converting enzyme (ACE2) (a key receptor for COVID-19) expression is higher in salivary glands than lungs, suggesting that these could be a major source of new viral particles.^33^ A recent study using mobility data and Bayesian inference inferred that a high rate of undocumented infections is responsible for rapid spread of SARS-CoV-2.^34^ Taken together, these data suggest that the potential for transmission is high early in the disease. While further studies are needed to better understand the relationship between viral load and symptom severity, it is expected that higher levels of viral shedding in the throat or lungs might be associated with an increased ability to infect others. To date, the relationship between lung and throat viral load in terms of disease severity, is not clear, and how dampening throat virus load may impact on resulting lung disease or viral transmission is not known.

The route of SARS-CoV-2 infection is currently considered to be via respiratory droplets, similar to SARS-CoV,^35^ and the virus particle is viable in aerosols for up to 3 h.^36^ Although we do not yet know the minimal infectious dose, the high rate of transmission indicates this is likely to be relatively low. If correct, then strategies to reduce the number of infective virus particles in mucous membranes through promoting their removal or inactivation could contribute to reducing risk of transmission. Thus, assuming that the throat is a major site of replication in early stages (even before symptoms are apparent), the oral washing using agents that could damage or destroy the lipid envelope has the potential to reduce viral load in the oropharynx.

At this time, there is incomplete information on how SARS-CoV-2 moves from the throat and nose to the lungs, and this could include (1) viral shedding, (2) the aspiration of necrotic cell debris, or (3) direct infection of neighboring cells. Assuming viral shedding is involved, the oral rinses that target the viral lipid envelope represent a potential method to remove/rinse or inactivate infective particles generated in the throat. The specific intracellular replication cycle for SARS-CoV-2 in humans is not yet known. Based on nonsynchronized replication cycles that take <24 h, virus is likely to be secreted almost constantly.^37^^,^^38^ Oral agents will impact only on virus that is extracellular or actively budding. Therefore, the persistence of treatment will be important. How long mouthwash components retain an ability to interact with biomembranes in the mouth is unclear, and more research is required.

## The Impact of Lower Ethanol Concentration on Biomembranes

When considering lower (nontoxic, more economical) ethanol concentrations, the literature on mammalian cells (from where the lipid envelope originates) provides a close comparator. We also reviewed studies on model membrane vesicles comprising phospholipids such as phosphatidylcholine; however, as these are protein-free, the impact of nonlipid components on ethanol toxicity is not accounted for. Bacterial pathogens contain very different membranes in lipid and protein composition, including lipopolysaccharides and peptidoglycans, so they are not considered here. Below we summarize the literature on impact of ethanol on cell/model membranes (Table 1).

**Table 1.** In vitro and in vivo data supporting the effects of ethanol on biomembranes or enveloped viruses

Reference	Study type	Ethanol	Results
Ly and Longo^39^	Model membrane vesicles (membrane fluidity, permeability, interdigitation, thickness, etc.)	Ethanol >3.4 M (20% v:v)	Membranes not considered “stable”; interdigitation; rapid swelling of PC vesicles
Ahl et al.^40^	Formation of *interdigitated* PL sheets from SUV	Ethanol above 2 M (11.8% v:v)	Formed larger IFVs; leakage of contents of vesicles
Hunt et al.^41^	Repeated cycling through transition phase of model membranes	Ethanol 86 mM (0.5% v:v)	Lysis of PC vesicles
Komatsu et al.^42–44^	Leakage of dye from vesicles made of PC, PE/PC, or PC/cholesterol.	0.6–2.1 M (3.5%–12.3%, v/v)	Calcein leaks out at low ethanol concentrations. Rapid swelling of vesicles.
Dennison et al.^45^	In vitro—Herpes, influenza, rotavirus, and adenovirus	26.9% ethanol (v:v) with essential oils	Enveloped viruses (herpes and influenza) were significantly impacted
IADR abstract 2010	H1N1 Influenza A pandemic strain, in vitro	21.6% ethanol, 30-s rinse	>99.99% reduction in infectivity
Roberts and Lloyd^46^	Three enveloped viruses: Sindbis, herpes simplex-1 and vaccinia, in vitro	20% (v:v) ethanol	Completely inactivated
Siddharta et al.^47^	Enveloped viruses; in vitro infectivity WHO formulation I in the presence of coronavirus.	30-s exposure of a dilution containing 34% (v:v) ethanol	Completely prevented subsequent viral replication
Oh et al.^48^	Mammalian cell membranes: Corneal epithelial cells	20% ethanol; 30-s incubation	40% loss of viability; high level of leakage of intracellular contents
Sonmez et al.^49^	Mammalian cell membranes: Red blood cells	1M (5.9% v:v) ethanol	Approximately 10% cell lysis
Chi and Wu^50^ and Tyulina et al.^51^^,^^52^	Mammalian cell membranes: Red blood cells	Moderate concentrations around 3–4M (18%–23.5%).	Potassium leakage and hemolysis
Wang et al.^53^	Mammalian cell membranes: Intestinal cell line (Caco-2)	Ethanol >5%–10%: long incubation time of 60 min	Loss of viability, leakage of contents, and disruption of tight junctions
Meiller et al.^54^	In vivo human study	21.6% ethanol, 30-s rinse	Recoverable virions of herpes simplex types I and II to 0 post rinse; at 30 min all lower than prerinse, 11/20 remained 0
Meiller et al.^54^	In vivo human repeat study	21.6% ethanol, 30-s rinse	0 recoverable virions in 18/20 post rinse and 12/20 at 30 min; at 60 min all less than baseline
Sattar et al. (unpublished data)	Finger pads of adults; Dried inocula; human respiratory coronavirus 229E	Hand gels with 60% and 70% ethanol exposed for 20 s	Viability titer of the virus was reduced by >99.99% in both cases

Studies cited in our text are summarized above for type, ethanol amount, and outcome. They are listed in order of model membranes, followed by in vitro studies on viruses, studies on mammalian cell membranes, then in vivo studies. Ethanol concentrations were listed also, in some cases, whether v/v or w/v was used was not provided in the study. In all studies, refer to the primary literature for full information on the impact of ethanol on the membrane. PC, Phosphatidylcholine, PE, phosphatidylethanolamine.

### Low Concentrations of Ethanol Cause Swelling, Interdigitation, and Leakage in Model Membranes

Biophysical studies in the 1980s and 1990s compared various alcohols (ethanol, methanol, butanol, and propanol) for their ability to perturb model phospholipid membranes. Many were optimizing generation of lipid vesicles for drug delivery; however, the toxicity of most short-chain alcohols prohibits oral use. Here, we reviewed reports on the properties of ethanol on model membranes. Few studies directly investigated lysis, instead focusing on membrane fluidity, permeability, interdigitation, thickness, and other parameters. In one study, ethanol >3.4 M (20% v/v) resulted in membranes not being considered “stable”.^39^ Ethanol addition causes rapid swelling of phosphatidylcholine vesicles from around 30 nm diameter at 0 M, up to between 80 and 110 nm diameter, at 1.1–1.5 M (6.5%–8.8% v/v).^55^*Interdigitation* refers to the process whereby the presence of short-chain alcohols enables the methyl group of the fatty acyl chains to move beyond the midplane of the bilayer, penetrating the opposite monolayer, and appears to be an event that precedes and promotes vesicle fusion and leakage.^56^^,^^57^ Several studies demonstrate that ethanol promotes interdigitation.^58^ In one, ethanol at above 2 M (11.8% v/v) led to formation of *interdigitated* phospholipid sheets from small unilamellar vesicles (SUVs), which then annealed to form larger interdigitation-fusion vesicles (IFVs).^40^ This means that ethanol at this concentration can deform small phospholipid vesicles leading to fusion and formation of larger structures. During this process, leakage of contents from vesicles is seen.^40^^,^^42^^,^^55^^,^^59^ Three studies compared membranes consisting of either phosphatidylcholine alone, phosphatidylcholine/phosphatidylethanolamine mixtures, or phosphatidylcholine/cholesterol mixtures, and showed that all became permeable at ethanol concentrations around 0.6–2.1 M (3.5%–12.3%, v/v).^43^^,^^44^^,^^55^ Elsewhere, ethanol at rather lower concentrations of 86 mM (0.5% v/v) caused lysis of phosphatidylcholine vesicles during repeated cycling through phase transition temperatures.^41^ Partitioning of ethanol into the membrane can be altered through the presence of additional biologically relevant lipid species such as cholesterol or gangliosides.^43^^,^^60^^,^^61^ This indicates that complex biological membranes may respond very differently, and not only the presence of other lipid types, but also the impact of proteins need to be taken into account. Nonetheless, it is clear that model membranes are sensitive to ethanol at concentrations far lower than the 60%–70% currently recommended for inactivation of virus on hard surfaces, and at amounts contained in widely available mouthwashes (Section 6).

### Impact of Ethanol on Mammalian Cell Membranes

We also reviewed the impact of ethanol on mammalian cells in vitro. Of direct relevance to coronaviruses, a study on corneal epithelial cells showed that a 30-s incubation with 20% ethanol led to around 40% loss of viability, which increased to 70% loss at 40% ethanol. There was significant leakage of intracellular contents following 20% ethanol for 30 s.^48^ This short incubation also altered inflammatory responses, differentiation, and epithelial marker expression.^48^ Several studies on the impact of ethanol on red blood cells were also found. Sonmez et al. showed that around 1 M (5.9% v/v) causes ∼10% cell lysis, but higher amounts were not tested.^49^ A variety of effects on red cells have been shown including potassium leakage and hemolysis, at moderate concentrations around 3–4 M (18%–23.5% v/v). However, incubation times of 15 min or greater were generally used.^50–52^ Last, a study on an intestinal cell line (Caco-2) showed that ethanol >5%–10% causes loss of viability, leakage of contents, and disruption of tight junctions, with a long incubation time of 60 min.^53^ Since the membrane composition of coronaviruses is expected to match ERGIC (Section 1), these studies provide strong evidence that low ethanol will directly impact on the SARS-CoV-2 membrane also.

So far, we found only studies that tested the impact of reduced ethanol amounts on enveloped viruses. Both were conducted in vitro, and show positive outcomes in relation to virus denaturation.

- In 2007, Roberts and Lloyd found that 20% ethanol completely inactivated three enveloped viruses: Sindbis, herpes simplex-1, and vaccinia, in vitro, while having no effect on the nonenveloped poliovirus-1.^46^ Inactivation was measured by inhibition of plaque-forming units in a viral infectivity assay, but direct impact on viral envelope was not determined. This study used a rather basic system, in the absence of a soil load, which is nowadays recommended under the American Society for Testing and Materials (ASTM) Committee E35 on Pesticides, Antimicrobials and Alternative Control Agents (https://www.astm.org/Standards/E2197.htm), or in the United Kingdom, the equivalent British Standard (BS) Norme Européenne (EN) standard (BS EN 14476:2013+A2:2019 Chemical disinfectants and antiseptics. Quantitative suspension test for the evaluation of virucidal activity in the medical area. Test method and requirements (Phase 2/Step 1), https://shop.bsigroup.com/ProductDetail?pid=000000000030401479). Also, it was conducted at 22°C, rather than the more relevant 36.8°C oral temperature, where the impact of denaturation agents would be greater.
- In 2017, Siddharta et al. tested WHO recommended formulations against enveloped viruses, including coronavirus. Focusing on WHO formulation I, which contains 85% (v/v) ethanol, 0.725% (v/v) glycerol, and 0.125% (v/v) hydrogen peroxide, they measured in vitro infectivity in the presence of a soil load (0.5% w/v bovine serum albumin). A 30-s exposure of a dilution containing 34% (v/v) ethanol (40% of neat) completely prevented subsequent viral replication.^47^

These studies indicate that relatively dilute ethanol will be highly effective against enveloped viruses. However, there is an urgent need to determine how coronaviruses are impacted by dilute alcohol under biologically relevant conditions (mucosa, mouth, etc.), and whether in combination with nontoxic, membrane disrupting agents, oral inactivation of SARS-CoV-2 could be achieved. A minimum amount of ethanol, for example, 10%–30% (v/v) would be effective, and contact time will also be an important parameter that may reduce ethanol exposure required. Ethanol impacts membrane properties of artificial lipid membranes, causing leakage of contents even in the absence of complete lysis. The ability of the virus to infect host cells could also be modified by inducing biophysical changes to the virus membrane which impact on protein function. The spike glycoprotein which is required for SARS-CoV-2 infectivity contains a transmembrane domain that is inserted into the viral envelope,^1^ and it is well known that lipid membrane biophysical perturbations can impact on conformation and function of many transmembrane proteins in mammalian cells. In this regard, the lipid membrane of HIV-1 was recently demonstrated to stabilize viral membrane glycoproteins and regulates their sensitivity to neutralization by antibodies.^62^ Thus, lower concentrations of ethanol could alter pathogenicity without complete neutralization of viral particles. Research is required to determine the impact of ethanol or other agents on the infective activity of the SARS-CoV-2 spike protein in vivo.

## Membrane Perturbation Without Lysis can Dampen Enveloped Virus Infectivity

The concept that perturbing the membrane could inactivate viruses has recently been tested in relation to membrane-disrupting agents, and an in vitro screen of 1000 compounds identified a series of lipidomimetics that can alter the membrane and dampen infectivity of HIV-1.^2^ Active agents included lipids related to cholesterol, sphingosine, or aliphatic lipids with long-chain fatty acids which blocked at the stage of entry into the host cell. The impact appeared to result from the lipids being incorporated into the membrane and inducing changes in lipid order and buoyant density of the particles. In support of this, a study in the 1970s showed that fatty acids and monoglycerides of 16–18 carbon chain length are highly effective in vitro, reducing survival of herpes simplex virus to around 50% at concentrations down to 0.2 μM.^63^ These studies use compounds that are nontoxic to mammalian cells and show great promise, thus research is needed to determine whether they are also active against the envelope of SARS-CoV-2 both in vitro and in vivo.

## Mouthwash Preparations that Show Activity Against Enveloped Viruses in Published Studies

We investigated the potential for commercially available mouthwashes to disrupt viral lipid envelopes, either due to ethanol (Table 1), or other active agents, through reviewing available literature.

Three industry-sponsored studies from the Universities of Maryland, Texas–Houston Health Sciences Centre, and State University New York tested this using a widely available formulation that combines 21%–26% ethanol with essential oils (eucalyptol 0.092%, menthol 0.042%, methyl salicylate 0.060%, and thymol 0.064% w/v). Notably, there is published evidence that eucalyptus oil and thymol have significant antiviral properties toward herpes simplex virus at these concentrations, hypothesized to relate to disruption of the viral lipid envelope.^64^

- The virucidal actions of 21% (v/v) ethanol with essential oils toward an enveloped virus were reported in humans in vivo in 2005. A 30-s rinse reduced infectious virions of herpes simplex types I and II to effectively zero.^54^ Specifically, 18/20 people demonstrated no virions postrinse, and after a 30-min rinse, virions remained at zero for 11/20 subjects, with all subjects remaining lower than prerinse levels. In contrast, rinsing with distilled water reduced mean virions considerably less post 30-s rinse, and levels had largely returned to baseline by 30 min. This indicates that the mouthwash had a specific and significant impact on virion recovery. In a repeat trial, 18/20 subjects had zero virions post 30-s rinse, with 12/20 remaining at zero at 30 min. At 60 min, all 20 were still shedding virions at 1–2 log_10_ lower than baseline, demonstrating a modest impact on viral titer. Longer contact times (eg, 60-s rinses) were not tested.^54^ Herpesviruses differ from coronaviruses in that the former can erupt periodically from where they reside in the nerves; so, using mouthwash may temporarily reduce the level and it may then help promote resolution of the lesion. On the contrary, coronaviruses will be shed almost constantly when actively replicating.
- A study in 1995 tested 26.9% ethanol with essential oils against herpes, influenza, rotavirus, and adenovirus in vitro. Here, an impact on the viral lipid envelope was speculated since herpes and influenza were significantly impacted, while adenovirus and rotavirus (nonenveloped) were not.^45^
- A follow-up unpublished study in 2010 determined that a 30-s in vitro exposure to 21.6% ethanol with essential oils led to >99.99% reduction of infectivity of H1N1 Influenza A pandemic strain (https://iadr.abstractarchives.com/abstract/2010dc-131191/evaluation-of-h1n1-antiviral-properties-of-an-essential-oil-containing-mouthrinse).

These studies provide proof-of-concept that mouthwashes containing essential oils with 21%–27% ethanol can inactivate enveloped viruses, both in the lab and in humans, with the likely mechanism being damage to the lipid envelope. Here, ethanol in combination with essential oils may provide a more effective formulation. Thus, these types of mouthwash may be effective against SARS-CoV-2, although studies have not been conducted. While other commercially available ethanol mouthwashes generally contain lower levels without essential oils, an impact on membrane biology may remain theoretically possible, and studies are required.

### Chlorhexidine

Chlorhexidine is widely used for oral health in the United Kingdom, being especially effective against Gram-positive bacteria, but to a lesser extent Gram-negative bacteria and fungi.^65^ Due to its positive charge, it reacts with the negatively charged microbial surface, penetrating into the cell and causing leakage. A report on its in vitro viricidal effectiveness at 0.12% has indicated it can reduce the viral concentration of enveloped but not nonenveloped viruses.^66^ However, this limited in vitro study only considered the immediate postexposure, and no further time points were included in the experimental design. Chlorhexidine is often formulated with ethanol at lower concentrations, which may in part explain its virucidal impact. A recent review of coronavirus literature identified that chlorhexidine exposure for 10 min only weakly inactivated coronavirus strains in suspension tests although the concentration used was low at 0.02%.^19^^,^^24^ Chlorhexidine formulations have been shown to retain oral antimicrobial activity for up to 12 h.^67^ It is a more effective antimicrobial in vivo because it binds to clean oral surfaces and is released over time (substantivity).^67^ Despite lower activity toward coronaviruses, a combination of chlorhexidine with alcohol may offer a useful strategy for reducing viral load over longer times.

Chlorhexidine mouthwashes have been a critical clinical tool for over 40 years to reduce oral bacterial flora and prevent infection and mucositis in cancer patients receiving chemotherapy and radiotherapy.^68–70^ However, there are no reported studies assessing the impact of mouthwashes in specifically preventing or treating viral infections in neutropenic patients. Last, a recent meta-analysis showed that chlorhexidine (rinse or gel) can reduce risk of ventilator-associated pneumonia in patients undergoing mechanical ventilation, although causative organisms were not described.^71^

### Povidone-Iodine

Povidone-iodine (PVP-I) mouthwash has been widely studied in relation to broad-spectrum antimicrobial and virucidal actions. At 0.23%, which is routinely used in Japan, this rapidly inactivates SARS-CoV, MERS-CoV, influenza virus A (H1N1), and rotavirus in vitro.^72^ A second study also showed that PVP-I (0.23%) is equivalent to 70% ethanol in inactivating SARS-CoV in vitro.^73^ Indeed, based on in vitro and limited clinical studies, in Japan, the Ministry of Health, Labour and Welfare supported daily gargling as a protective measure to prevent upper respiratory tract infections.^74^ A small number of human studies supporting this in the case of PVP-I have shown reduced incidence of both bacterial and viral (influenza) infection through repeated gargling.^72^^,^^75^ In one rather limited study, the absence rate in middle schools in Yamagata City was compared over 3 months, where PVP-I gargling was encouraged in 1 school, versus 7 where it was not. A reduction of absence due to colds and influenza from a mean of 25.5% (no gargling) down to 19.8% (*P* < 0.05) was found.^76^ In another study, a group of 23 patients gargled more than 4 times/day for up to 2 h. Here, acute exacerbation of chronic respiratory infection was reduced by around 50%.^75^ This mouthwash is not available in the United Kingdom, although may still be purchased in Germany and other countries. As a 1% solution, PVP-I is available in Hong Kong, Korea, Singapore, Malaysia, Philippines, and Taiwan. The importance of higher concentrations of PVP-I as a broad-spectrum antimicrobial agent for topical uses is indicated by its inclusion on the World Health Organization’s List of Essential Medicines (https://www.who.int/medicines/publications/essentialmedicines/en/). It should be noted that rare allergic reactions have been reported for PVP-I.^77^

### Chlorinated Water or Hypertonic Saline Rinsing

Studies from Japan surprisingly found that gargling with chlorinated tap water reduced respiratory infections, and in one, was even better than PVP-I. In one, three groups of around 130 age- and gender-matched human subjects were studied (three 15-s gargles with 20 mL, at least 3/day for 60 days).^78^ Tap water reduced incidence of common cold by 36%, while PVP-I was not effective. It was speculated that chlorine in the water may have contributed, since levels in Japan were above concentrations that are known to have viricidal activity including toward enveloped species.^79^ However, information on the virucidal impact of chlorine comes from in vitro studies, including 1 with a 30 min contact time, and its impact in vivo on enveloped viruses, through gargling tap water is not known.^79^ In 2008, another trial calculated economics of the activity in two groups of around 120 subjects gargling water for 60 days and concluded that this was a cost-effective strategy for upper respiratory infection prevention.^80^ Last, a recent study showed that gargling and nasal rinsing with hypertonic saline could reduce symptoms, duration of illness, and viral shedding. However, this was a pilot, nonblinded, self-reported study and so cannot be considered definitive.^81^ A follow-up study in vitro with enveloped and non/enveloped viruses including human coronavirus 229E suggested that this may have been related to altered intracellular chloride levels and peroxidase activities.^82^ None of the in vivo studies addressed the issue of which pathogens were contributing to illness, and so cannot be extrapolated to coronaviruses.

Separate to oral rinsing it is worth noting that nasal rinsing with saline is a popular method promoted to clear nasal passages for sufferers of colds and allergies. Given that virus is recovered in the nasopharynx, a similar consideration of how this might be used as preventative measure could be made. As for mouthwash, clinical studies have not systematically examined how effective nasal rinsing is for preventing respiratory infections. Notably, rare reports of serious illness when not properly cleaned, due to the presence of parasitic amoebae in unboiled tap water, has led to recommendations on careful disinfection of rinsing syringes being made by CDC (https://www.cdc.gov/parasites/naegleria/sinus-rinsing.html).

### Hydrogen Peroxide

Hydrogen peroxide causes oxygen-free radical-induced disruption of lipid membranes and is widely used as an agent for tooth whitening. Studies, including a recent systematic review, report that coronavirus 229E and other enveloped viruses are inactivated at concentrations around 0.5%.^19^^,^^83^ While higher concentrations of hydrogen peroxide (>5%) will induce damage to both soft and hard tissues, within the range of concentrations used in mouthwashes for whitening at 1%–3% little damage is reported.^84^ Within the oral environment, hydrogen peroxide is rapidly inactivated due to the presence of host- and bacteria-derived catalase activity in saliva and other endogenous peroxidases.^85^ The impact of peroxidases could theoretically be reduced by using a prerinse with water, although this is untested. A consideration with this agent is that it can have potential proviral activities, although so far this was only seen in vitro.^86–88^

### Quaternary ammonium compounds

These are widely used as microbicidal agents that interfere with protein or lipid components on the cell surface, particularly Gram-positive or Gram-negative bacteria. Their virucidal activities are not widely reported although some reports against enveloped viruses have been made in the literature relating to surface disinfection.^79^ Among this group of compounds, cetylpyridinium chloride (CPC) was recently shown to have activity against influenza both in vitro and in vivo, through direct attack on the viral envelope, with in vitro EC50 being 5–20 μg/mL.^89^ CPC is used in medicated oral rinses at concentrations 0.025%–0.075% w/v (250–750 µg/mL) in the United Kingdom, while lozenges sold in some countries contain 1.4–3.0 mg of CPC.

## Current Policies Relating to Oral Health and Use of Microbicides in Dental Practice and with Immunosuppressed Individuals

Dental practitioners are at elevated risk of exposure to SARS-CoV-2, and there are guidelines that advocate use of mouthwash clinically. Previously published CDC guidelines for infection control in the dental setting have cited the potential usefulness of preprocedural mouthwashes in reducing the spread of airborne pathogens of all types.^90^ Indeed, studies have addressed how virucidal components including CPC and chlorhexidine can be effective in reducing bacterial contamination in this setting, although virus inactivation was not tested.^91^^,^^92^ Meng et al. in an experience-based review of their practice in Wuhan, recommended preprocedural mouthwash to reduce the oral microbial load in patients undergoing dental treatment in patients with SARS-CoV-2.^93^ Last, two recent papers aimed at providing guidelines for endodontists in relation to SARS-CoV-2 advocated preprocedural mouth rinse with 0.2% PVP-I.^94^^,^^95^ Given that the dental community recognize, the potential for oral mouth washing in relation to reducing infection risk, extrapolating these guidelines to the wider community is worth a full discussion.

## The Urgent Need for Research

Many questions need to be addressed in relation to whether oral hygiene could represent a viable approach to dampen transmission of SARS-CoV-2, and research is required to address this.

In relation to oral hygiene, we need to determine:

- Can we reduce viral load in the oropharynx through oral rinsing?
- If we can reduce load, then which oral rinse would be clinically effective: The current choice includes 20%–30% ethanol, lipid-based membrane disruptors, PVP-1, CPC, hydrogen peroxide, simple chlorinated tap water or WHO formulation I diluted to 30% of neat?
- Would a combination of agents in lower amounts be better tolerated, reducing adverse effects, and remain effective?
- What combinations or agents, contact time and frequency of use would induce antiviral activity and reduce infectivity of SARS-CoV-2?

Available research approaches include:

*Statistical epidemiological studies* could establish on a population level whether mouth rinsing is associated with reduced rates of throat and respiratory infections including SARS-CoV-2. Purchasing data of health-related products to model health linkages could be used. New applications to conduct widespread monitoring of SARS-CoV-2 symptoms, could capture use of mouthwashes to test for correlation with symptoms and severity, alongside wider purchasing sales behavior of those who are asymptomatic. Modeling approaches should also consider population usage of mouthwash preparations and viral spread.
*Underpinning research*. Not all enveloped viruses are the same, and herpes, influenza, and measles viruses are considered more unstable than human coronaviruses, which may persist for up to 5 days on inanimate surfaces.^19^^,^^36^^,^^96^ Thus, research needs to focus on coronaviruses in particular. The exact composition of the SARS-CoV-2 lipidome needs to be determined using lipidomics mass spectrometry. Research should determine the impact of ethanol or other agents on the infective activity of the spike protein itself, in vitro, and in vivo. A useful virus to test in vitro would be the human respiratory coronavirus 229E which is used extensively as a surrogate for human coronaviruses but only requires Category (Cat2) procedures, and its replication and propagation conditions are well established already. This would be a good representative for pathogenic coronaviruses, prior to narrowing down to SARS-CoV-2 which requires Cat3 biosecurity.
The impact of temperature and soil load needs to be considered for in vitro studies, applying the ASTM or EU standard protocols (https://www.astm.org/Standards/E2197.htm). Here, the impact of not only dose/composition but also the critical issue of contact time with agents, which is a known modifiable parameter of virucidal activity can be easily tested. The virucidal mechanisms can be determined, conducting lipidomics analysis of the envelope along with assays that determine spike protein conformation and activity. While a particular ethanol concentration may achieve full inactivation, lower amounts could either help to remove virus or lead to membrane damage (permeability/leakage) that may impact throat cell virus infectivity, for example, through potential modification of the ability of the spike glycoprotein to interact with receptors on host cells. In this regard, for HIV-1, the lipid membrane stabilizes membrane glycoproteins, regulating their sensitivity to antibody neutralization.^62^ This type of action could be further enhanced if membrane disrupting agents were also included in a mouthwash. Indeed, lipidomimetic compounds have already been developed that can dampen viral infection through affecting lipid membrane structure or curvature (discussed in Section “Membrane Perturbation Without Lysis can Dampen Enveloped Virus Infectivity”).
Most virucidal research uses in vitro models, where the response to the agent will be different, and also does not take into account the impact of host immunity. There is an absence of animal model studies on coronavirus respiratory illness, although macaques and mice transgenic for humanized ACE2 are beginning to be studied with early indications being that both develop mild illnesses in response to SARS-CoV-2 virus (https://www.nature.com/articles/d41586-020-00698-x).
*Clinical studies*. Robustly designed, appropriately powered in vivo clinical studies are needed, including determination of the most effective composition. Self-reported, nonrandomized, unblinded studies are not reliable and need to be avoided. An important sequela of these over the counter medicines is that individuals may use them prior to providing diagnostic nasopharyngeal/throat swabs. This could increase the number of false-negative tests and facilitate transmission. Currently, there is no specific advice to avoid these preparations prior to testing.
*Population-based interventions* could be considered, although panic buying or dangerous consumption of ethanol or methanol has to be avoided. As high-risk groups come out of self-isolation, they could represent a population to evaluate clinical outcomes resulting from real-world use of available mouthwashes. The current social restrictions will reduce a number of transmission risk factor variables and alter clinical outcomes in terms of SARS-CoV-2 infection, other respiratory infections, and adverse effects, but monitoring outcomes could provide useful data. Users could be given general advice on product use, recording timings, and duration of gargling for later analysis, or act as controls. Similarly, health workers at high risk of infection could be provided products and asked to record their use and report outcomes. Ideally, throat swabs and blood samples would be obtained for testing. There would be logistical, ethical, and regulatory issues involved in setting up investigations. However, given the theoretical plausibility and data we have reviewed plus the readily available products and urgent need to reduce SARS-CoV-2 infection, measures could be considered and action taken to instigate clinical investigation in the population during the outbreak.
*Host inflammation*. Mouthwashes widely utilized in daily oral and dental hygiene for cosmetic and medical reasons and have demonstrated acceptable tolerability when used multiple times daily for durations of 6 months and longer. Despite this, the impact of rinsing with these agents on throat tissue health needs to be seriously considered, since the viral lipid membrane is effectively the same as that of the host. Some of these agents, such as ethanol and hydrogen peroxide may, if used several times a day over a period of 2–3 months, induce mucosal inflammation. This was observed in a study on corneal epithelial cells, where inflammatory cytokines (IL-1b, IL-6), chemokines (IL-8/CXCL8, CCL2), and matrix metalloproteases (MMP9) were all upregulated at the mRNA levels 1–3 days after a 30-s exposure to 20% (v/v) ethanol.^48^ Here, it will be important to ascertain whether a repeated daily rinse with mouthwash would have any detrimental impact on the stromal tissue lining. Alternatively, host innate immune responses in early infection could also represent a strategy to remove virus, and this has not been considered in any studies to date. Significant advances have been made into the molecular basis of alcohol-induced tissue injury. However, these studies tend to be confined to studies of acute and chronic alcohol consumption where the metabolism of alcohol into acetaldehyde and reactive oxygen intermediates modify various physiological processes linked with the maintenance of tissue homeostasis.^97^ Currently, there is a lack of research into the potential impact of mouthwash on local inflammation within the throat and consideration needs be given to both its impact on antiviral immunity and the disruption of tissue integrity.

### Additional Reading

We highlight a list of excellent review articles that were consulted as part of preparing this review and were a source of primary research cited herein:

Budding of viral lipid membranes: Simons and Garoff^98^

Impact of virus on lipid metabolism: Sanchez and Lagunoff^99^

Review of inactivation of coronaviruses on surfaces: Wolff et al.^100^

Emergence of SARS-CoV in 2003: Peiris et al.^101^

Two papers on the impact of ethanol on interdigitation of membranes: Slatter and Huang^102^^,^^103^

Control of infection using Povidone-Iodine: Eggers^104^

Summary of the composition of lipid membranes in cells: van Meer^105^

### Search strategy

Since the review covers many areas from basic biochemistry, virology, and microbicidal research, as well as clinical information both in medicine and dentistry, multiple sources were consulted. Most references were identified from PubMed, ResearchGate, or Google, using search terms including “virus,” “coronavirus,” “lipid envelope,” “alcohol,” “membrane,” “chlorhexidine,” and others, alone and in combination. Many references that were first identified, were then investigated further to find additional source material and the original primary research which was then included. The idea for drafting the review was initiated by V.B.O. on March 21, 2020, through reaching out in person to various international experts, to get their views and input directly, via phone calls and then follow-up emails. Boots UK was approached initially to discuss the ideas and for information on formulation of available mouthwashes. Boots researchers and scientists contacted Johnson & Johnson, who provided proprietary information. Boots researchers (Kirkdale, Thornley, Povey, Inchley, and O’Shea) then conducted further searches of PubMed, Google, and ResearchGate, using the same terms. Academic and clinical expertise was consulted for virology (Stanton, Humphreys, and Bosch), clinical/ICU (Fegan and Wise), dental practice (Thomas), immunology (Jones), lipid biochemistry (Wakelem, Murphy, Simons, and O’Donnell), and microbicides (Sattar and Maillard). Phone/email correspondence with experts generated input, opinions, and identified additional references. No timeline for references was used and no languages were excluded.
